# The efficacy of artificial dermis combined with continuous vacuum sealing drainage in deep neck multiple spaces infection treatment

**DOI:** 10.1097/MD.0000000000024367

**Published:** 2021-02-05

**Authors:** Xiang Gu, Wei Chen, Kun Yuan, Jian Tan, Suguang Sun

**Affiliations:** Department of Otolaryngology, Head and Neck Surgery, The Central Hospital of Wuhan, Tongji Medical College, Huazhong University of Science and Technology, Wuhan, China.

**Keywords:** efficacies analysis, multiple spaces infection, the neck abscess, vacuum sealing drainage

## Abstract

Deep neck abscesses are dangerous. Artificial dermis combined with seal negative pressure drainage is a new technique for treating refractory wounds.

To compare the efficacy of vacuum sealing drainage (VSD) with that of traditional incision drainage approaches for treating deep neck multiple spaces infections.

This retrospective analysis includes patient data from our hospital collected from January 2010 to March 2020. A total of 20 cases were identified. Based on the treatment methods, the patients were divided into the VSD group and the traditional group. Inflammation indicators (white blood count, WBC), duration of antibiotic use, hospitalization time, doctors’ workload (frequency of dressing changes) and treatment cost were analyzed and compared between the two groups.

Of the 20 patients, 11 patients underwent treatment with VSD, while the other 9 underwent traditional treatment. All patients were cured after treatment. Compared with the traditional group, the VSD group had a slower decline in the inflammation index, shorter duration of antibiotic use, shorter hospital stay, and lower doctor workloads (*P* < .001). There was no significant difference in treatment cost between the two groups (*P* > .05).

VSD technology can markedly improve the therapeutic effect of deep neck multiple spaces infection. This treatment method can be used to rapidly control infections and is valuable in the clinic (*P* > .05).

## 1 Introduction

Deep neck multiple spaces abscessed form when two or more head and neck fascia spaces are infected. The rapid development of the infections can cause serious complications. Some infections may be life-threatening, such as those that cause airway obstruction, jugular vein thrombosis, descending mediastinitis, sepsis, acute respiratory distress syndrome, and disseminated intravascular coagulation. Deep neck abscesses are dangerous.^[1–3]^ The early application of antibiotics can control or partially cure neck infections.^[4–6]^ Therefore, there has been a decline in the incidence of this type of disease in recent years. Surgical incision combined with debridement and drainage has been the preferred treatment for neck multiple spaces infections that are difficult to control with antibiotics.^[7]^ However, clinicians must address persistent problems, such as burdensome dressing changes, long-term and mass antibiotic use, prolonged infection and long-time hospital stays. Artificial dermis combined with seal negative pressure drainage is a new technique for treating refractory wounds. The formation of closed negative pressure in the infected area can eliminate the dead space, resulting in the timely removal of exudate and necrotic tissue, promoting the growth of granulation tissue and accelerating wound healing. Instead of the rear application in the treatment of neck multiple spaces infection, vacuum sealing drainage (VSD) is commonly used in burns, large areas of cutaneous defects, stress-induced injury, diabetic foot ulcer, etc.^[8–11]^ This paper retrospectively studied data from 20 patients with deep neck multiple spaces infection in our hospital and compared the efficacy of VSD with that of traditional treatment for reducing the occurrence of postoperative complications due to deep neck multiple spaces abscess and improving the postoperative comprehensive benefits among patients. All patients included in this study provided informed consent and agreed to the use of their medical history information. The ethics committee of The Central Hospital of Wuhan, Tongji Medical College, Huazhong University of Science and Technology for the data of the patients approved the study. All works were undertaken following the provisions of the Declaration of Helsinki.

## 2 Materials and methods

### 2.1 Study design

This paper retrospectively analyzed data from patients with deep neck multiple spaces infections at The Central Hospital of Wuhan. The data were collected from January 2010 to March 2020. Based on the treatment methods, the patients were divided into two groups. The traditional group included 9 patients (6 males, 3 females) with an average age of 60.2 ± 11.3; these patients were treated with antibiotics and traditional incision debridement drainage. The VSD group included 11 patients (6 males, 5 females) with an average age of 57.1 ± 10.2; these patients were treated with antibiotics and VSD. The inclusion criteria were as follows:

1) neck space infections of various causes;
2) abscess was indicated in preoperative specialist examination, neck ultrasound or CT;
3) patients with basic diseases aggravated by infections (such as electrolyte disorders, heart or kidney insufficiency);
4) patients with symptoms, such as sepsis, bacteremia, or septic shock.

The exclusion criteria were as follows:

1) patients with incomplete clinical data;
2) patients with severe diabetes and severe renal failure.

During the course of the disease, most patients had clinical symptoms, such as fever, local swelling, difficulty opening their mouth, pain from swallowing, etc. There was no significant difference in gender, age or disease condition between the two groups (*P* > .05), indicating comparability. All cases were examined by blood routine, biochemistry, neck CT or MRI, etc., to observe the infection area. The efficacy of VSD was compared to that of traditional treatment. The preoperative and postoperative 3-day inflammatory index (WBC), duration of antibiotics, length of hospital stay, workload of doctors (frequency of dressing change) and treatment costs were assessed.

### 2.2 Therapeutic methods

In the traditional group, the wound was repeatedly rinsed with normal saline, hydrogen peroxide and iodophor after the abscess cavity was thoroughly cleared and the necrotic tissue was removed. Then, a drainage tube was placed in the abscess cavity for drainage. The patient was given a local rinse and dressing change. The frequency of the dressing change was adjusted until there was no obvious pus exudation in the operative area. When the wound surface had fresh granulation tissue and no obvious purulent exudation, we waited for self-healing or sutured after the tension reduced.

**Figure 1:**
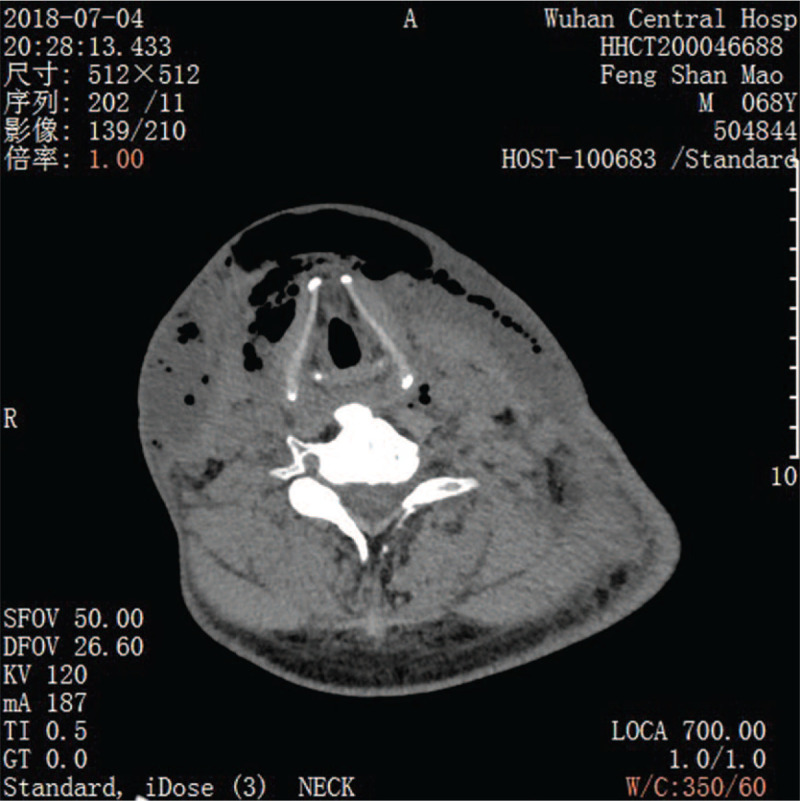
Preoperative CT examination (axial) in the vacuum sealing drainage-assisted irrigation group.

**Figure 2:**
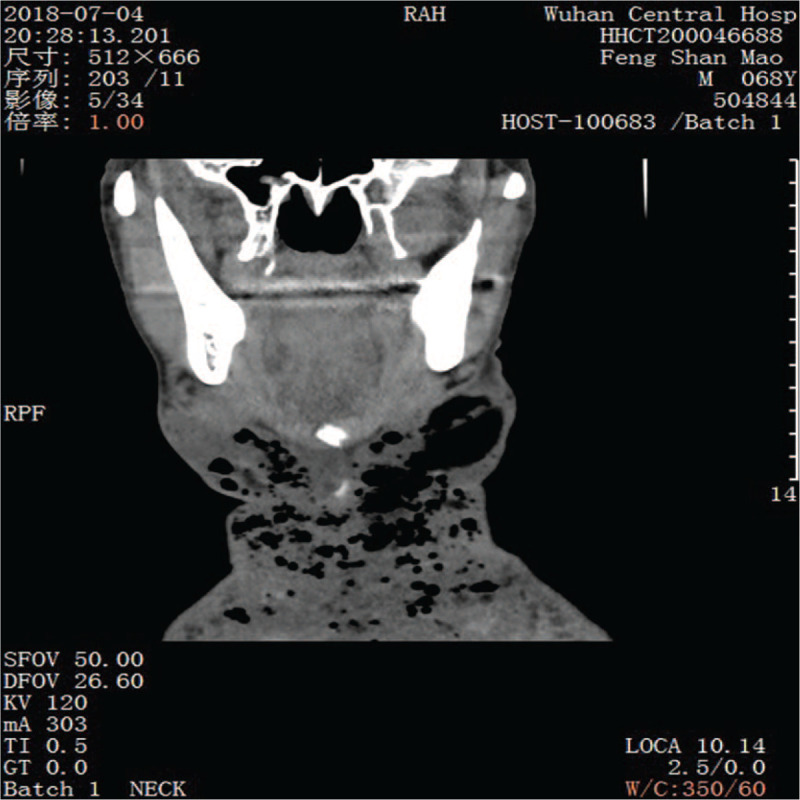
Preoperative CT examination (coronal) in the vacuum sealing drainage-assisted irrigation group.

**Figure 3:**
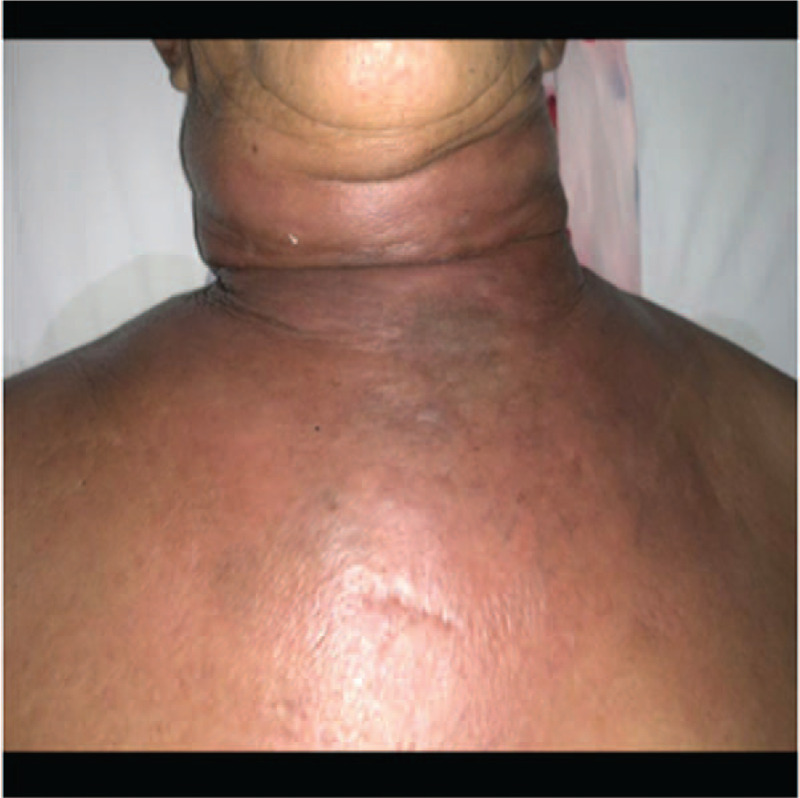
Extensive cellulitis of the neck, tension, obvious tenderness and fluctuation in palpation.

**Figure 4:**
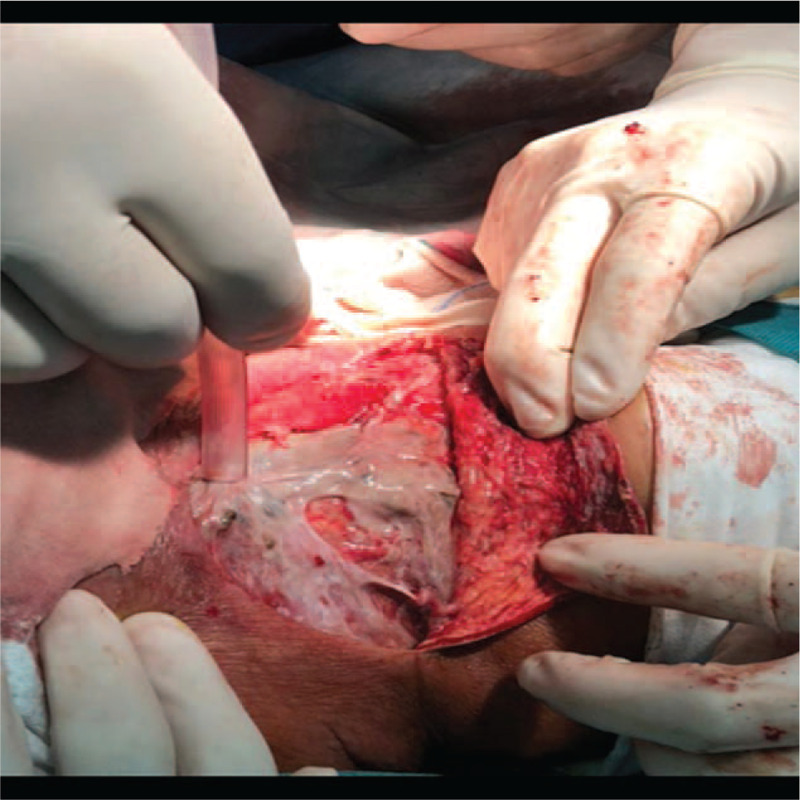
A large amount of necrotic pseudo membrane.

**Figure 5:**
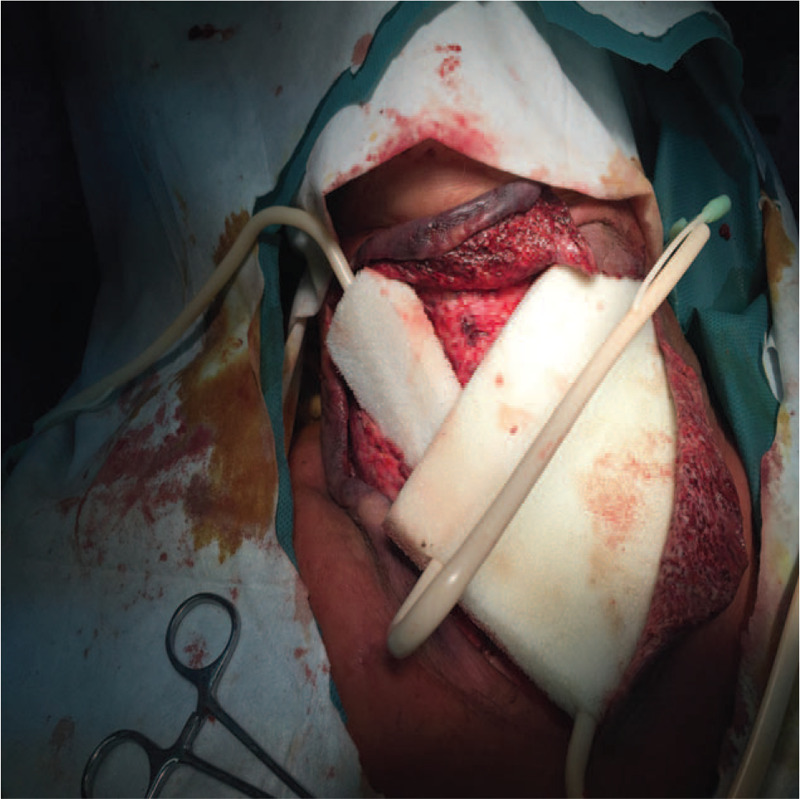
VSD drainage was implanted.

**Figure 6:**
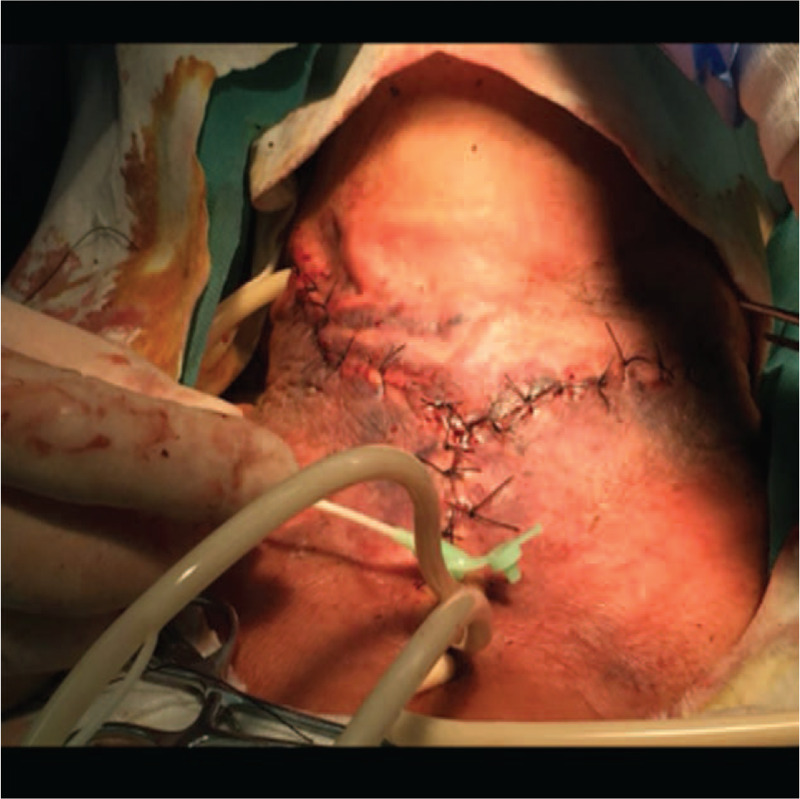
Placement of sponge and tight closure of wound cavity.

**Figure 7:**
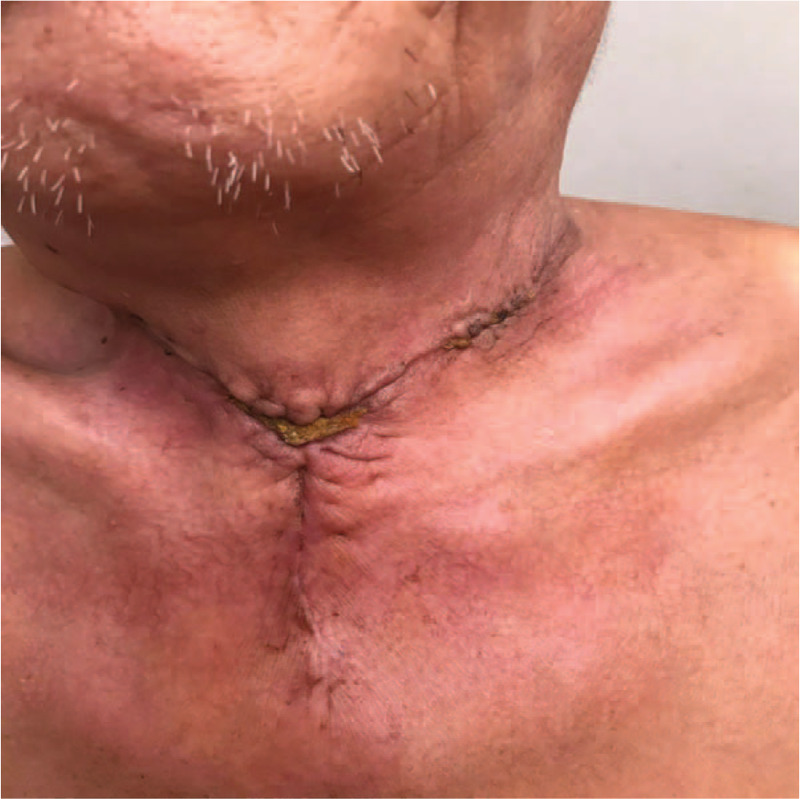
Healing status of the patient 1 month after surgery.

In the VSD group, the patients underwent color Doppler ultrasonography or spiral computed tomography (CT), including axial (Fig. 1) and coronal (Fig. 2) directions, to identify abscess sites and ranges. Extensive cellulitis of the neck, obvious tenderness and fluctuation in palpation were observed (Fig. 3). The skin of the patients was incised at the sites of redness and swelling or at the sites of the intense fluctuation, and after incision and discharge of pus, a large amount of necrotic pseudo membrane was observed (Fig. 4). After debridement and drainage, the VSD material was trimmed according to the shape of the wound and implanted to cover the wound (Fig. 5). An irrigation tube and a drainage tube were placed in the high vacuum material to seal the wound tightly, and the wound cavity was closed (Fig. 6). The daily lavage and extraction volume were recorded postoperatively. The VSD was removed 5 to 7 days later. If there was no necrotic pseudo membrane during the operation and the wound was covered with fresh granulation tissue and had no pus or exudation, a suture could be considered. If the infection still existed, another VSD membrane could be considered. After the symptoms and related indicators improved, the vacuum drainage kit was removed, and the wound was sutured. The observed healing status of the patient 1 month after surgery was documented (Fig. 7).

The 2 groups underwent bacterial culture and drug sensitivity tests and were treated with sensitive antibiotics. Inflammatory indicators, duration of antibiotic use, hospitalization time, doctors’ workload (frequency of dressing change) and treatment cost were analyzed and compared between the two groups.

### 2.3 Statistical methods

SPSS 23.0 statistical software was used to calculate and analyze the data of this study. Categorical data was analyzed using the χ^2^ test; quantitative data were presented as the mean ± standard deviation (x ± s), and the t-test was used for between-group comparisons. A *P* < .05 was considered statistically significant.

## 3 Results

All patients underwent pus culture and antimicrobial susceptibility test, among which streptococcus (7 patients) and staphylococcus (5 patients) were the most commonly observed pathogenic bacteria. The type of antibiotics was adjusted based on the results of bacterial culture. The most commonly used antibiotics were β-lactamase inhibitors. According to postoperative examinations, 2 patients were diagnosed with branchial cleft cysts, and 2 patients were diagnosed with acute salivary adenitis. Three patients suffered from tracheal intubation due to airway obstruction or swelling of the throat, which was life-threatening. Emergency tracheotomy was performed to open the airway under local anesthesia. Through comparing the inflammatory indicators, the degree of improvement in the VSD group was significantly better than that in the traditional group (*P* < .001). The workload of doctors was evaluated based on the frequency of dressing changes and was significantly lower in the VSD group than in the traditional group (*P* < .001). Additionally, the hospitalization time in the VSD group was shorter than that in the conventional group (*P* < .001). The cost of required materials was higher than that of the traditional group, while the cost of dressing change was higher and the duration of antibiotic use was longer in the traditional group. There was no significant difference in the treatment cost between the two groups (*P* > .05) (Table 1). Postoperative follow-up showed no recurrence.

**Table 1:** Comparison of results between the two treatment modalities.

Group	n	WBC (before operation)	WBC (postoperation 3 days)	Cure duration (d)	Dressing change (frequency)	Duration of antibiotic use (d)	Cost of treatment (CNY)
VSD	11	12.52 ± 0.84	6.84 ± 0.66	12.27 ± 1.10	3.82 ± 0.75	10.45 ± 1.57	47745.45 ± 4377.98
traditional	9	12.23 ± 0.89	9.13 ± 0.84	17.67 ± 1.50	17.89 ± 1.27	16.11 ± 1.05	46300.00 ± 3303.03
*t*		0.762	−6.826	−9.268	−30.858	−9.209	0.817
*P*		.456	<.001	<.001	<.001	<.001	.425

## 4 Discussion

The spaces involved in the deep neck multiple spaces abscess mainly include the submandibular space, the parapharyngeal space, the carotid space, the retropharyngeal space, the parotid space, the anterior cervical space, and the pretracheal space. If two or more are involved, then it is defined as a ductile space. Deep neck abscess is a severe but treatable infection characterized by rapid progression and life-threatening complications.^[12,13]^ In our cases, the most common clinical symptoms are sore throat and neck swelling. The submandibular space and the anterior neck space are the most frequently involved spaces. Dental and upper respiratory tract infections are the main causes. Airway obstruction is one of the important complications. Tracheotomy is still an effective method to ensure postoperative safety and plays an important role in reducing complications and improving prognosis.^[14,15]^ Bacterial culture of pus presents more negative results, which may be related to the early application of antibiotics, anaerobic bacteria are not cultured, and improper sampling of materials and other factors, so the bacteriological culture is only used as a reference for antibiotics. As the fascia spaces in the neck are interconnected, if improperly controlled, local infections tend to spread rapidly and cause multiple spaces infections. Therefore, early identification and active treatment are crucial to prevent adverse consequences. After the abscess is opened, putrefactive and necrotic bacteria can still form a large amount of purulent secretion in the deep part of the neck. Methods for addressing the drainage and the subsequent purulent cavity cleaning should be considered in the treatment of such diseases.^[2,16]^ The traditional treatment method is to expand the abscess cavity, reduce the local pressure, flush and discharge the abscess, and change dressings frequently after surgery. Dressing changes 1–2 times a day after surgery will consolidate the burden of the clinicians and the psychological burden and pain of the patients.

VSD involves placing the drainage tube into polyvinyl alcohol foam and discharging the exudate through the drainage tube by negative pressure suction. Compared with the traditional method, VSD can make the irrigation more sufficient. The everyday routine involves observing the condition of the wound and the drainage of negative pressure. No dressing is required if nothing is abnormal. One week later, the VSD material can be removed. VSD greatly reduces the workload of the staff.

Artificial dermis combined seal negative pressure drainage will integrate the dressing and the negative pressure drainage, enabling the physiological saline to finish the rinse and drainage of pus cavity surfaces several times in a day, forming a closed drainage system for the wound, promoting wound drainage, removing exudate from the drainage area, and rapidly providing the wound a clean and stable internal environment. This method will reduce dressing change frequency, accelerate the growth of granulation tissue, and reduce the burden for clinicians. In addition, compared with irrigating and changing dressings twice a day in the traditional method, there is less pain caused by negative pressure irrigation and drainage.

This paper and follow-up study showed that the level of white blood cell decline in the VSD group was significantly lower than that in the traditional group, indicating that VSD plays an important role in accelerating the recovery from inflammation. Studies in molecular biology have shown that long-term local negative pressure can stimulate micro angiogenesis around the wound, increase local blood circulation, and provide more phagocytes and antibody components for the wound. This fact contributes to the defense function of the immune system and improves the local anti-inflammatory capacity.^[17,18]^ VSD can reduce the inflammatory reaction, accelerate epithelial regeneration, and promote the alignment of collagen fibers. VSD promotes angiogenesis by increasing the quantity and quality of collagen.^[19]^ Additionally, VSD can increase the expression of transforming growth factor β1 (TGF-β1), which will promote granulation tissue growth and contribute to wound healing.^[20]^ The duration of antibiotic use in the VSD group was lower than in the traditional group. The Defined Daily Dose system (DDDs) was significantly reduced, as was the long-term use of antibiotics in large doses, thus lowering the chance of drug-resistant bacterial infection. In conclusion, VSD provides an effective treatment for the recovery of neck infection and is a safe and reliable surgical method. However, there are also some shortcomings in its clinical application, such as the increasing economic burden of patients due to the use of high-value consumables and the potential need for a second operation to remove the implanted artificial dermis as there is a certain risk of air leakage and insufficient drainage.

We hence draw the following conclusions, which are conducive to the clinical application of this method. VSD eliminates the dead space and the occurrence of reinfection by extensively expanding the wound during surgery, accelerating the drainage, and removing the necrotic pseudo membrane sufficiently. During the operation, the necrotic lymph nodes should be confirmed by disease examination to avoid the possibility of metastatic cancer. For extremely severe neck infections, in some cases, the artificial dermis must be reimplanted and several debridement operations must occur to confirm that the granulation tissue grows well. Then, the wound is considered closed. During the surgery, attention should be paid to avoid injury to the large vessels of the neck, which may cause serious consequences. Adjusting the infusion rate on the irrigation side and the negative pressure drainage force on the drainage side to make the pressure relatively balanced will not only improve the irrigation efficiency but also avoid pipe blockage caused by excessive negative pressure. Wound surface and drainage should be carefully managed. Information, such as irrigation, volume, character and color, should be recorded. The main reason for the failure of drainage is that blood clots, pus or debris block the drainage tube. If a blood clot is suspected, the drainage tube can be squeezed up and down. If the blood clot or exudate blocks the drainage tube, an empty needle can be used for suction, 20 ml sterile saline can be injected to rinse the catheter, or streptokinase or trypsin can be injected to dissolve the blood clot to facilitate drainage. If the infected patients are nutritionally deficient, negative pressure drainage is more likely to lead to hypoproteinemia. Relevant indicators should be closely monitored to provide nutritional support and prevent related complications. For the case of deep neck multiple spaces infection, we believe that VSD has unique advantages and great practicability to comprehensively benefit patients in the clinic.

Although the incidence of deep neck multiple spaces infections is declining yearly, it should still be taken seriously. Tracheotomies for patients with acute laryngeal obstruction must be performed, and there may be difficulty in tracheal intubation restricted by mouth opening and difficulty breathing after abscess incision and drainage. Ageing and associated systemic disease can cause serious complications. In addition, if the infection cannot be controlled within 24 to 48 h during treatment, abscess incision and drainage should be performed in a timely manner. A negative pus culture result does not mean that there is no bacterial growth, and the location and time of the material are important. The use of antibiotics sensitive to streptococcus can be used as an empirical treatment plan. Imagological examination is also extremely important for the development of individualized treatment strategies. Due to the low incidence of deep neck multiple spaces infection and the small number of samples, it is difficult to perform prospective research, so additional multicenter large-sample studies are needed. In future cases, we will further discuss certain topics, such as how to shorten the treatment cycle and determine the cleanliness of the wound and hyperbaric oxygen adjuvant therapy.

VSD technology can improve the treatment of deep neck multiple spaces infection, is conducive to the rapid control of infection, and is valuable in the clinic.

## Author contributions

**Conceptualization:** Xiang Gu.

**Data curation:** Xiang Gu, Suguang Sun.

**Formal analysis:** Xiang Gu, Suguang Sun.

**Investigation:** Jian Tan.

**Methodology:** Xiang Gu, Kun Yuan.

**Resources:** Suguang Sun.

**Supervision:** Wei Chen.

**Writing – original draft:** Xiang Gu, Wei Chen.

**Writing – review & editing:** Suguang Sun.
